# HAL® exoskeleton training improves walking parameters and normalizes cortical excitability in primary somatosensory cortex in spinal cord injury patients

**DOI:** 10.1186/s12984-015-0058-9

**Published:** 2015-08-20

**Authors:** Matthias Sczesny-Kaiser, Oliver Höffken, Mirko Aach, Oliver Cruciger, Dennis Grasmücke, Renate Meindl, Thomas A. Schildhauer, Peter Schwenkreis, Martin Tegenthoff

**Affiliations:** Department of Neurology, BG University Hospital Bergmannsheil Bochum, Ruhr-University Bochum, Bürkle-de-la-Camp-Platz 1, 44789 Bochum, Germany; Department of Spinal Cord Injuries, BG University Hospital Bergmannsheil Bochum, Ruhr-University Bochum, Bürkle-de-la-Camp-Platz 1, 44789 Bochum, Germany; Department of General and Trauma Surgery, BG University Hospital Bergmannsheil, Ruhr-University Bochum, Bürkle-de-la-Camp-Platz 1, 44789 Bochum, Germany

## Abstract

**Background:**

Reorganization in the sensorimotor cortex accompanied by increased excitability and enlarged body representations is a consequence of spinal cord injury (SCI). Robotic-assisted bodyweight supported treadmill training (BWSTT) was hypothesized to induce reorganization and improve walking function.

**Objective:**

To assess whether BWSTT with hybrid assistive limb® (HAL®) exoskeleton affects cortical excitability in the primary somatosensory cortex (S1) in SCI patients, as measured by paired-pulse somatosensory evoked potentials (ppSEP) stimulated above the level of injury.

**Methods:**

Eleven SCI patients took part in HAL® assisted BWSTT for 3 months. PpSEP were conducted before and after this training period, where the amplitude ratios (SEP amplitude following double pulses - SEP amplitude following single pulses) were assessed and compared to eleven healthy control subjects. To assess improvement in walking function, we used the 10-m walk test, timed-up-and-go test, the 6-min walk test, and the lower extremity motor score.

**Results:**

PpSEPs were significantly increased in SCI patients as compared to controls at baseline. Following training, ppSEPs were increased from baseline and no longer significantly differed from controls. Walking parameters also showed significant improvements, yet there was no significant correlation between ppSEP measures and walking parameters.

**Conclusions:**

The findings suggest that robotic-assisted BWSTT with HAL® in SCI patients is capable of inducing cortical plasticity following highly repetitive, active locomotive use of paretic legs. While there was no significant correlation of excitability with walking parameters, brain areas other than S1 might reflect improvement of walking functions. EEG and neuroimaging studies may provide further information about supraspinal plastic processes and foci in SCI rehabilitation.

## Background

Following spinal cord injury (SCI), substantial functional and structural reorganization can be observed in primary somatosensory (S1) and primary motor (M1) cortices [1–7]. Following SCI, deafferentiation of both sensory afferents and motor efferents results in enlarged cortical maps and increased excitability in sensorimotor cortical areas representing the intact limbs proximal to the spinal lesion [1, 4, 8]. Transcranial magnetic stimulation (TMS), a technique that induces targeted cortical activation, has been used to test cortical excitability and to map cortical areas of the motor cortex. With this technique, it has been shown that SCI patients have increased cortical excitability and an enlarged cortical representation associated with muscles proximal to the level of the spinal cord lesion [2, 4, 9]. Functional magnetic resonance imaging (fMRI) studies in paraplegic patients support these results, showing an enlarged body representation with increased and a medially-shifted, i.e., towards the leg representation, activation maxima in cortical areas related to muscles proximal to the lesion [5, 6, 8, 10]. These changes likely reflect neuronal plastic mechanisms in the primary motor cortex (M1), which are necessary to adapt to physical impairments and to optimize walking abilities [11]. The pathological changes following SCI are not restricted to corticospinal tract, however, but also extend to sensory pathways and the primary somatosensory cortex. Using electrical cortical mapping in nonhuman primates with upper limb deafferentiation, for example, Pons et al. observed a medial shift of face representation into the area of the deafferentiated limb [12]. In the human S1, *Henderson et al.* similarly observed functional and cerebral structural reorganization in SCI patients [6], such that the fifth digit representation in SCI patients was enlarged and had shifted medially toward the cortical area associated with the sensory loss. This functional reorganization was accompanied by structural changes in these patients, where new intracortical lateral connections were formed [6]. Together these results demonstrate important supraspinal plastic effects of motor and somatosensory pathways and identify cortical areas directly involved in cortical reorganization following SCI. It is still unclear, however, if there is also an alteration in S1 excitability following SCI, as seen previously in M1. Furthermore, it is unclear whether the processes are affected by therapeutic interventions, such as intensive walking and increased use of the impaired extremities. Therefore, we investigated how HAL®-assisted BWSTT may alter both behavioral measures and S1 excitability in paraplegic SCI patients.

Cortical excitability can be measured by means of so-called paired-pulse stimulation techniques of evoked potentials [13–17]. By providing two electrical pulses to the skin in short succession, a notable reduction in the amplitude of the evoked potential measured from S1, can be observed following the second pulse, as compared to the S1 response to single pulse. This suppressive effect of the second stimulus has been consistently measured in healthy subjects and is taken as a measure of excitability. Low paired-pulse suppression, is reported by high amplitude ratios and is indicative of high cortical excitability, whereas high suppression, is reported by low amplitude ratios and is indicative of low cortical excitability [17, 18]. The commonly investigated amplitude N20P25 of both evoked responses are measured and expressed as a ratio (“amplitude ratio”). The N20P25 complex is determined as the difference between the N20 peak and the peak of the subsequent positivity P25. It is reliable amplitude that represents the cortical response in S1. Paired-pulse SEPs have been used to assess excitability effects in healthy humans [18, 19], as well as in neurological impaired states [20] as well as in perceptual learning [17, 21]. Excitability measurements of S1 have not been yet employed, however, in rehabilitation medicine, and specifically in SCI patients, to assess training effects. While various training techniques are used clinically, exoskeletons provide assistance to the patient, encouraging proper re-learning of various movements (e.g., walking) while providing needed support. Little is known about plastic effects of different SCI rehabilitation-training methods like manual or robotic-assisted bodyweight supported treadmill training (BWSTT), however the latter has been shown to enhance functional locomotion in SCI patients [22]. In general, plasticity mechanisms that are associated with recovery of function are upregulation of neurotrophins, including brain derived neurotrophic factor (BDNF), reduction of inhibitory spinal excitability, and training-dependent changes of cortical excitability and cortical maps [23–26].

For robotic assistance, various exoskeletons are available. In contrast to other exoskeletons, HAL® (hybrid assistive limb®) exoskeleton (Cyberdyne Inc., Japan) offers the possibility of monitoring muscle contractions via surface EMG-electrodes at the extensor-flexor muscle region of the lower extremities [27, 28]. This allows for voluntary machine-supported motion using minimal signals recorded from hip and knee flexors and extensors. In both a pilot study and a single case study, we demonstrated that BWSTT with HAL® exoskeleton in SCI patients resulted in improved functional abilities for over-ground walking, as measured by the 10-m walk test (10MWT), 6-min walk test (6MWT) and timed-up-and-go test (TUG test) [28, 29]. Voluntary drive and normalized motion assistance provided by the external device forms the foundation for a proprioceptive feedback loop for patients with lesions involving sensory pathways. The neural activity and repeated execution of specific tasks promote learning and leads to the reinstatement or restructuring of appropriate proprioceptive feedback [30, 31]. This mechanism explains the therapeutic effect of locomotor training using HAL® [32]. So far, there are no studies investigating possible plastic effects on walking due to HAL® training.

Here, we examined reorganization as a consequence of HAL®-assisted BWSTT in paraplegic SCI patients. First, we hypothesized that paraplegic SCI patients would have an increased excitability of the hand area in S1, similar to the observations in M1, as a consequence of cortical plastic effects following SCI. Second, based on the fact that proprioceptive feedback induces plasticity [31, 33], we postulate that plastic changes in S1 following SCI could be partly reversed by intensive, active, voluntary-driven and robotic-assisted walking training with HAL® exoskeleton. Furthermore, we hypothesized that changes in S1 excitability would correlate with improvements in walking parameters. To measure cortical excitability, we used paired-pulse somatosensory evoked potentials of median nerves, as mentioned above [17]. Understanding neuronal plastic effects following exoskeletal training with HAL® is of importance, as it could demonstrate the clinical potential of locomotor training with an exoskeleton.

## Methods

### Subjects

For this pilot study, we enrolled 11 patients (four females, seven males). Clinical data are presented in Table 1. Only functional data of cases 1–7 were published previously in our pilot study [28]. Neither functional nor electrophysiological data of cases 8 to 11 have been published yet. Mean age at the time of training start was 46.9 +/− 2.7 years SEM (standard error of the mean). The mean time since injury was 8.8 +/− 2.1 years. Inclusion criteria were traumatic SCI with incomplete paraplegia or complete paraplegia after lesions of the conus medullaris/ cauda equina with zones of partial preservation. Patients were classified according to the American Spinal Injury Association Impairment Scale (ASIA A/B/C/D) [34]. Independent of ASIA classification the enrolled patients were required to present motor functions of hip and knee extensor and flexor muscle groups in order to be able to trigger the exoskeleton. Exclusion criteria were as follows: non traumatic SCI, pressure sores, severe limitation of range of motion regarding hip and knee joints, cognitive impairment, body weight > 100 kg, non-consolidated fractures and moderate or severe heart insufficiency. In order to compare excitability levels, we collected ppSEP data from 11 healthy subjects for control group (mean age 27.8 +/− 2.7 years SEM). All control subjects were free of medication. The study was approved by Ethical Board Committee of Ruhr University of Bochum and followed the Declaration of Helsinki. All patients provided written informed consent.

**Table 1:** Clinical data of SCI patients

Case	Sex	Age (in years)	Time since injury (in years)	Etiology	Level	ASIA classification/ZPP	Current medication
1	M	40	13	T7/8#	T8	C	Tolterodine 4 mg/day
2	M	63	1	T12#	L1	B/L3	Trospium chloride (unknown dosage)
3	M	36	1	T11/12#	T12	A/L3	Oxybutynin 20 mg/day
4	F	55	1	L1#	L1	C	Thyroxine 75 μg/day, pregabalin 150 mg/day, methionine 1500 mg/day
5	M	42	16	L1#	L1	A/L3	Oxybutynin 15 mg/day, methionine 1500 mg/day
6	M	52	10	L3#	L2	A/L3	Methionine 1000 mg/day, calcium (unknown dosage), cranberry capsules 2/day, alendronic acid 70 mg/once a week
7	F	40	19	L1#	T11	A/S1	N0ne
8	M	56	0.7	L1#	T12	C	Trospium chloride 30 mg/day, amlodipine 5 mg/day, domperidone 10 mg/day
			(8.5 months)				
9	F	36	8	L1#	L1	A/L3	None
10	M	52	10	L1#	L1	C	Ramipril 5 mg/day, amlodipine 5 mg/day, oxybutynin 20 mg/day
11	F	44	17	L1#	L1	C	None

*M* = male, *f* = female, # = fracture, *ASIA* = American Spinal Injury Association, *ZPP* = zone of partial preservation, *T* = thoracic, *L* = lumbal, *S* = sacral

### The exoskeletal training

**Fig. 1:**
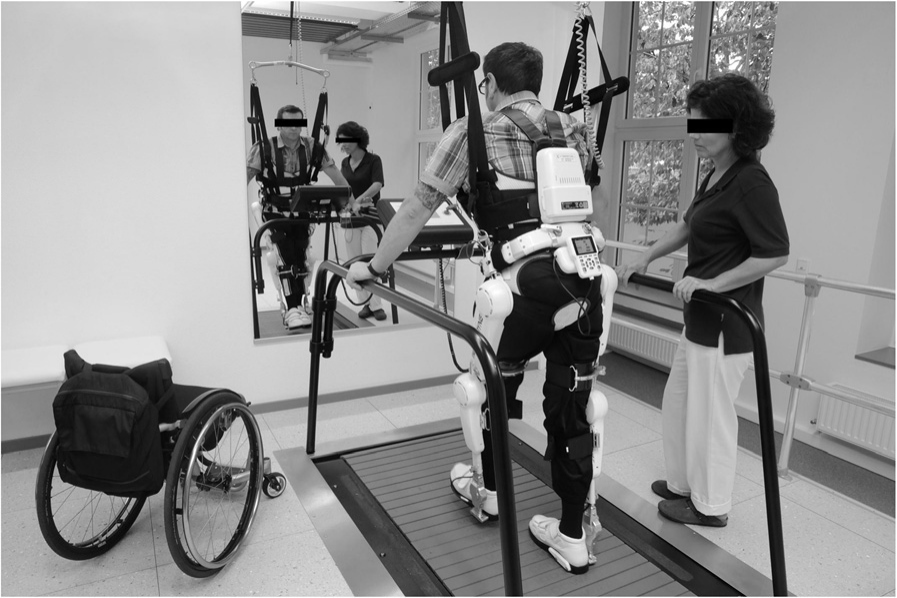
Hybrid assistive limb® (HAL®). Bodyweight supported treadmill training with the HAL® exoskeleton. (Copyright V. Daum, Bergmannsheil Bochum)

All patients underwent a 3-month training period of BWSTT with the HAL® exoskeleton. Each patient was scheduled for a 30 min training session 5 times a week for 12 weeks, as previously described by our group [28]. HAL® is an exoskeleton with a frame and robotic actuators that attach to the patient’s legs. The joint movement is supported by electric motors. Voluntarily-initiated minimal bioelectrical signals measured from extensor and flexor muscles of hip and knee are detected via surface EMG electrodes. Through a cable connection between exoskeleton and patient, this system allows voluntary robotic-supported movements (Fig. 1). The treadmill system (Woodway USA, Inc., Waukesha, WI, USA) includes a body weight support system with a harness. The speed can be adjusted from 0 km/h to approximately 4.5 km/h. During treatment, the velocity of the treadmill was set individually between comfortable and maximum speed tolerated by the patient. Initially, the harness system supported approximately 50 % of each patient’s body weight. This was individually reduced in subsequent training sessions, as tolerated without substantial knee buckling or toe drag. No adverse events occurred during the intervention.

### Study design

All subjects were trained with BWSTT assisted by HAL® for 12 weeks, preceded and followed by electrophysiological measurements. The pre-training testing was done within 2 days prior to the start of training. Post-training testing was done 1 day following the completion of the 12-week training period. No other walking therapy or additional physical therapy was given to these patients during the study. To compare excitability levels, we performed ppSEP measurements on healthy controls.

### Paired-pulse SEP of median nerves

**Fig. 2:**
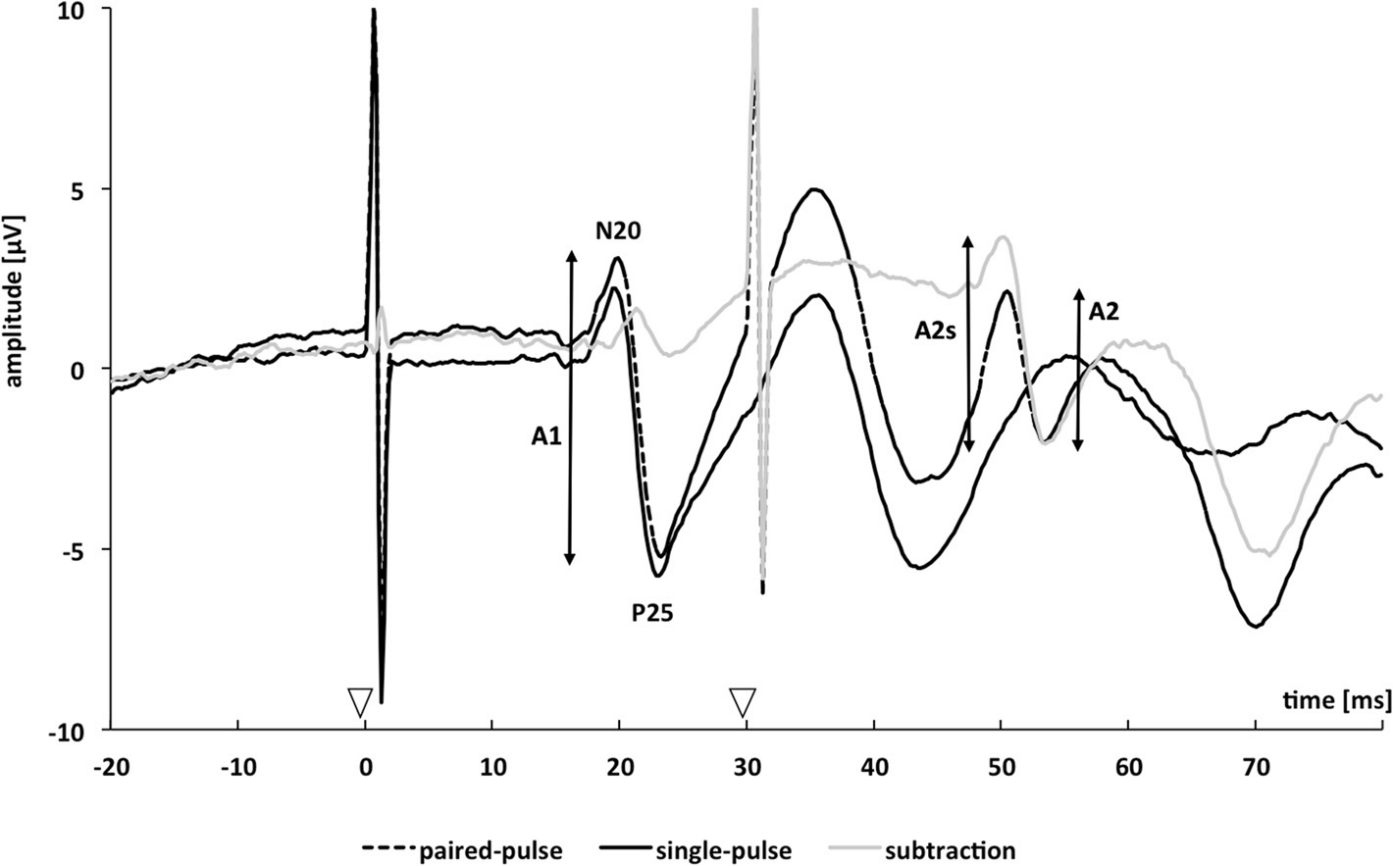
Paired-pulse somatosensory evoked potentials. Somatosensory evoked potentials over cortical CP3 or CP4 of one subject after single (continuous black trace) and paired-pulse stimulation with interstimulus interval of 30 ms (continuous grey trace). The triangles along the x-axis represent the applied electrical stimuli. The dotted black trace results by subtracting the single-pulse trace from the paired-pulse trace. The analyzed amplitudes of the first response (A1) and second response (A2) after paired-pulse stimulation are marked by vertical bars; amplitudes of the second response after subtracting the response to a single pulse are denoted as A2s

Before and after the entire HAL®-training period and once for the control group, we applied single and paired electrical stimulation of the median nerves with an interstimulus interval of 30 ms in combination with recordings of somatosensory evoked potentials (SEP). Nerve stimulation was performed consecutively on both sides with a block electrode placed on the wrist (pulse duration 0.2 ms, repetitive rate of the stimuli 3 Hz). Subjects had to report a prickling sensation in the thumb, index and middle finger of the stimulated hand to verify correct positioning of the stimulating block electrode. Stimulus strength was adjusted to 2.5 times the perception threshold [18]. In all participants, the chosen stimulation intensity induced a small muscular twitch in the thenar muscles. During median nerve stimulation and SEP recordings, subjects were seated in a comfortable chair and were instructed to relax but stay awake with closed eyes. SEPs were recorded and stored for offline analysis with a 32-channel-amplifier (Brain Amp, Brain Products, Germany), with a sampling rate of 5 kHz and band-pass filtering between 2 and 1000Hz). SEP recordings were made using an eight-electrode array. Electrodes were placed over CP3 and CP4 (primary somatosensory cortex, according to the international 10–20 system) [35]. A reference electrode was placed over the midfront (F_Z_) position. Offline, a total number of 800 evoked potentials after single and paired-pulse stimulation of both sides each were recorded in epochs from 30 before and 150 ms after the stimulus and averaged. Evoked potentials were provided sequentially (i. e. single-pulse left, single-pulse right, paired-pulse left, paired-pulse right). Peak-to-peak amplitudes after single and paired-pulse stimulation of the N20-P25 over S1 response component were analyzed. As shown in Fig. 2, following paired-pulse stimulation the response to the second pulse rides on the response to the first pulse, leading to a superimposition of both evoked potentials. Therefore, the amplitude of the response to the second pulse may misleadingly appear to be increased or decreased. To assess the “true” paired-pulse interaction, linear superposition effects were factored out by subtracting the response to single pulse stimulation from the response to paired-pulse stimulation trace. We analyzed the amplitude of the response to the second stimulus of the paired-pulse stimulation after linear subtraction of the response to single pulse stimulation (second amplitude after subtraction = A2s) and referred it to the response to the first stimulus of the paired-pulse stimulation before linear subtraction (A1). Paired-pulse suppression was expressed as a ratio (A2s/A1) of the amplitudes of the second (A2s) and the first (A1) peaks (see Fig. 2) and was our primary outcome parameter. For correlation analysis, we calculated the difference of mean amplitude ratios “post-pre” (ΔA2s/A1_post-pre_).

**Fig. 3:**
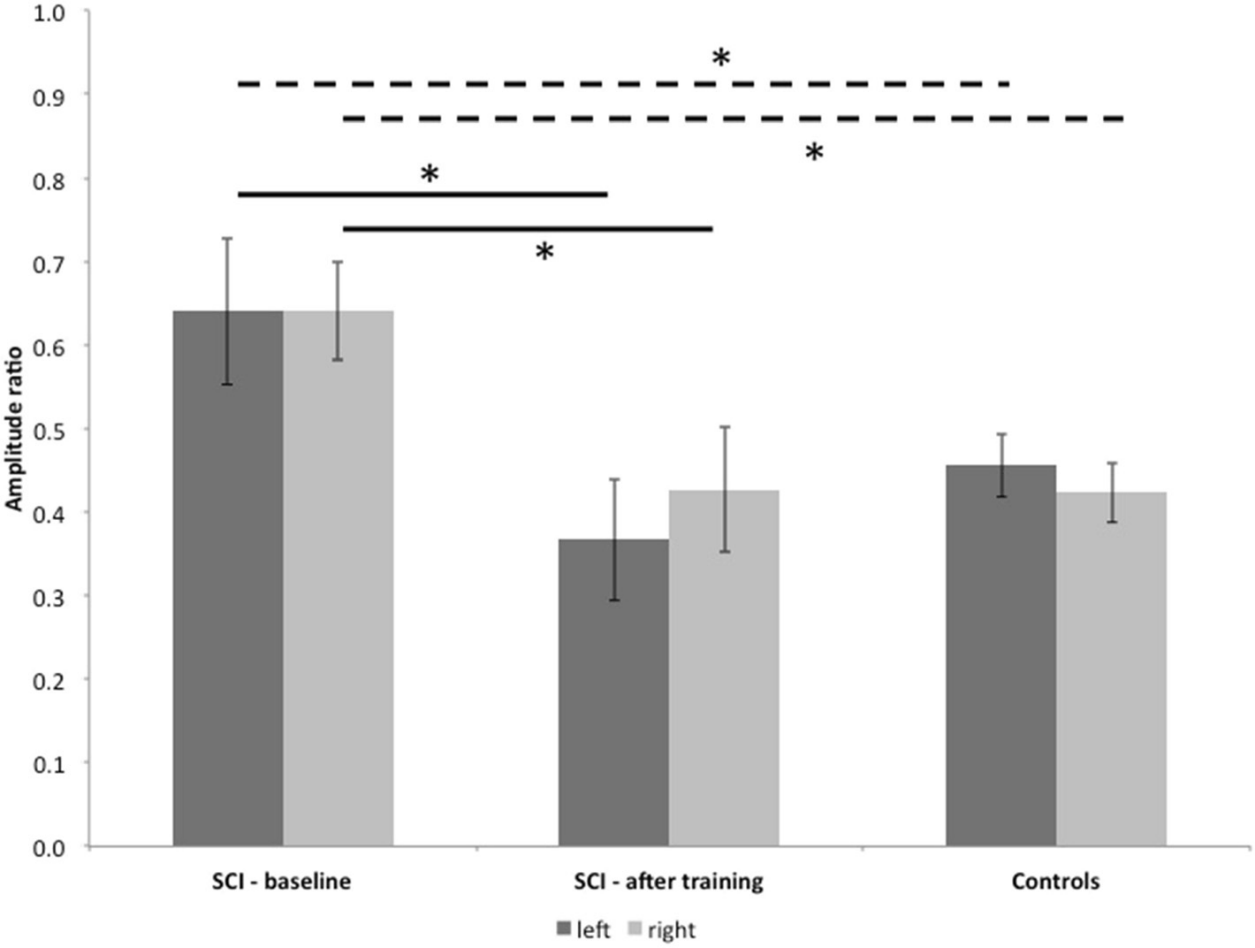
Paired-pulse suppression in spinal cord injury. The bars present mean amplitude ratios (A2s/A1) before and after the training period and of healthy controls. Error bars indicate standard error of the mean. * = significant *p* < 0.05. SCI = spinal cord injury

### ENG, SEP of tibial nerves and MEP

Electroneurography, somatosensory evoked potentials of the tibial nerves, and motor evoked potentials (MEP) of anterior tibial muscles were performed before and after the training period. All examinations were conducted with a Neuropack M1 (Nihon Kohden, Tokyo, Japan). Nerve conduction studies included compound muscle action potentials (CMAP), distal motor latencies and motor nerve conduction velocities (mCV) of the right peroneal and left tibial nerves, as well as sensory nerve action potentials (SNAP) and sensory nerve conduction velocities (sCV) of right sural and left peroneal nerves. All stimulations were supramaximal. Standardized protocols were used for all measurements. SEPs of tibial nerves were recorded over C_Z_ (cortical representation) with a reference placed over F_z_, according to standardized protocol for evoked potentials [36]. MEPs were measured simultaneously via surface EMG-electrodes from both anterior tibial muscles. A double cone coil was used for simultaneous stimulation of both hemispheres [37]. MEP-amplitude and MEP-latency were measured. To assess the peripheral motor conduction time (PMCT), we used the *F-wave* method with electrical stimulation of the peroneal nerve at the level of the caput fibulae [37]. M-response latency, M-response amplitude and F-wave latency were analyzed. Central motor conduction time (CMCT) and PMCT were calculated by following formula:

$$ \mathrm{A}\Big)\mathrm{PMCT}=\left(\mathrm{M}\hbox{-} \mathrm{responselatency}+\mathrm{F}\hbox{-} \mathrm{wavelatency}\hbox{-} 1\right)/2 $$

$$ \mathrm{B}\Big)\mathrm{CMCT}=\mathrm{M}\mathrm{E}\mathrm{P}\hbox{-} \mathrm{latency}\hbox{-} \mathrm{P}\mathrm{M}\mathrm{C}\mathrm{T} $$

Furthermore, we calculated the relative MEP-amplitude (MEP-amplitude/M-response amplitude x 100).

### Functional measurements

The 10MWT was done before and after each training session. It detected the time, the number of steps, and the required assistance to walk a 10 m distance [38, 39]. The TUG test describes the time and assistance required for standing up from the wheelchair, walk 3 m, turn around, walk back and sit down [38, 40]. It was performed every 2 weeks. The 6MWT was done at the beginning, at half time and at the end if possible, depending on the patient. It evaluates the distance while walking for 6 min [38, 41]. The neurological status was assessed using the ASIA Impairment Scale modified from the Frankel classification and classifies motor and sensory impairments that result from a SCI [34, 42]. The lower extremity motor score (LEMS) in this study was obtained by the addition of the impairment scores (0–5) of the lower extremity key muscles of both sides.

### Statistical analysis

Student’s paired two-sided *t*-tests were used to analyze differences between both stimulation sides at baseline or after training, differences between amplitude ratios before and after the HAL® exoskeleton training period and N20-latencies of the single pulse and first response of the paired pulse. Furthermore, we analyzed differences of amplitude ratios between SCI patients and healthy controls before and after training (Student’s unpaired two-sided *t*-test). To assess training improvement, functional measurements (10MWT, 6MWT, TUG) and the LEMS were analyzed by Student’s paired two-sided *t*-test also. Significance was assumed at the 5 % alpha level. Statistical calculations were performed using the IBM SPSS Statistics 22.0 software package. In order to assess linear correlations between change of amplitude ratio (ΔA2s/A1_post-pre_) and functional parameters, LEMS and time since injury, we performed linear bivariate correlation analysis (two- tailed Pearson’s correlation).

## Results

### Paired-pulse SEP of median nerves

There was a statistical difference between SCI-patients and healthy controls at baseline (SCI: left: 0.64 +/− 0.06 SEM, right: 0.64 +/− 0.07; healthy controls: left: 0.51 +/− 0.05, right: 0.46 +/− 0.05; left: *p* = 0.042, right: *p* = 0.022). Looking at the training effect, analysis showed a significant statistical training effect in the patients group (SCI pre: left: 0.64 +/− 0.06 SEM, right: 0.64 +/− 0.07; SCI post: left: 0.37 +/− 0.07, right: 0.43 +/− 0.07; left: *p* = 0.003, right: *p* = 0.015). Comparing the amplitude ratios between patients post training and healthy controls, there was no statistical significant difference (SCI post: left: 0.37 +/− 0.07, right: 0.43 +/− 0.07; healthy controls: left: 0.51 +/− 0.05, right: 0.46 +/− 0.05; left: *p* = 0.216, right: *p* = 0.685). Results are shown in Fig. 3.

To rule out that altered amplitude ratios after training were caused by a reduction of the first response of paired-pulses (A1, see in Fig. 1), we compared the amplitudes of first response of paired-pulses (N20-P25). We found no significant difference after the training on both sides (left: *p* = 0.279, right: *p* = 0.385). Additionally, the latencies of the single pulse N20 and the N20-amplitude of first response of paired pulse were analyzed. For no condition, a significant effect on latencies was found. For further statistical correlation analysis, we calculated the mean amplitude ratio of each stimulation side and the post-pre-difference, expressed as ΔA2s/A1_post-pre_.

### Functional parameters and LEM-score

Table 3 sums up the functional walking parameters, LEM-scores and amplitude ratios. There were statistical significant differences between pre and post measures in SCI patients in 10MWT-speed (pre: 0.25 m/s +/− 0.05 SEM, post: 0.5 m/s +/− 0.07 SEM, *p* = 0.001), 6MWT (pre: 86 m +/− 20.86 SEM, post: 149.73 m +/− 20.32 SEM, *p* < 0.001), TUG test (pre: 56.35 s +/− 10.06 SEM, post: 38.65 s +/− 7.2 SEM, *p* = 0.01) and LEM-score (pre: 21.27 +/− 2 SEM, post: 24.36 +/− 2.08, *p* = 0.001). We calculated the post-pre differences to perform correlation analysis.

### ENG, SEP of tibial nerves and MEP

In summary, no significant differences after the training period could be detected. Due to cauda equina lesions, most of the conduction studies, SEPs and MEPs revealed conduction blocks. Table 2 sums up the results. Because of the resulting small dataset, we calculated median instead of mean values all measures.

**Table 2:** Results of ENG, SEP of tibial nerves and MEP. Difference of parameters before and after the training period

	Pre-post difference		Pre-post difference	
Electroneurography	CMAP [mV]	Conduction velocity (motor) [m/s]	SNAP [μV]	Conduction velocity (sensory) [m/s]
*Peroneal nerve, right (motor)*	−1.7	3.0		
*Peroneal nerve, left (sensory)*			−4	−2
*Tibial nerve, left (motor)*	0.8	3.0		
*Sural nerve, right*			6.0	1
SEP of tibial nerve	P40 [ms]	P_1_N_2_ [μV]		
Left	−0.8/ NR = 8	−0.1/ NR = 8		
Right	−0.8/ NR−8	−0.1/ NR = 8		
MEP anterior tibial muscles	CMCT [ms]	PMCT [ms]		
	1 patient had contraindication (aneurysma coil); for all other patients CMCT and PMCT could not be calculated due to highgrade lesion or conduction block (missing MEP or M-response or F-wave)			

Left/right, *NR* = no response, *CMAP* = compound muscle action potential, *SNAP* = sensory nerve action potential, *SEP* = somatosensory evoked potential, *MEP* = motor evoked potential, *CMCT* = central motor conduction time, *PMCT* = peripheral motor conduction time

### Correlation analysis

Linear bivariate analysis between mean ΔA2s/A1_post-pre_and time since injury did not reveal significant correlation (*r* = 0.65, *p* = 0.85). Table 3 contains correlation analysis between excitability and functional parameters. We did not find any significant correlations.

**Table 3:** Functional and excitability data. Table shows the training effect (post-pre differences) and statistical analysis. Correlation analysis between mean amplitude ratio and functional parameter. Statistical significance level *p* < 0.05

Case	Mean amplitude ratio	10MWT speed (m/s)	6MWT distance (m)	TUG test (s)	LEMS
1	−0.51	0.23	38	−5.57	2
2	0.04	0.04	28	1.17	8
3	−0.40	0.13	81	−8,75	2
4	−0.12	0.41	59	−57.09	2
5	−0.09	0.41	60	−14.37	3
6	−0.10	0.10	68	−34	2
7	−0.20	0.30	63	−21.54	1
8	−0.52	0.41	85	−4.16	5
9	−0.28	0.47	66	−12.67	6
10	−0.27	0.26	149	1.8	2
11	−0.25	0.02	4	−39.56	1
Mean value	−0.24	0.25	63.72	−17.70	3
SEM	0.05	0.05	11.11	5.64	0.68
*p*-value	*na*	0.001	<0.001	0.01	0.001
Correlation factor *r* (*p*-value)		−0.002 (ns)	−0.221 (ns)	−0.318 (ns)	0.214 (ns)

*10MWT* = 10-m walk test, *6MWT* = 6-min walk test*, TUG test* = timed-up and go test, *LEMS* = lower-motor-extremity score, *SEM* = standard error of the mean, *na* = not applicable

## Discussion

In this study we investigated effects of the HAL® exoskeleton training on behavioral and electrophysiological measures of clinical severity in SCI patients. Here we show that SCI patients had increased cortical excitability in the hand area of S1 prior to training and that this was normalized following 12 weeks of BWSTT with HAL® exoskeleton. These changes following training were accompanied by significant improvements in walking abilities without HAL® exoskeleton for over-ground walking measured by the 10 MWT, 6MWT and TUG test. ENG, SEP of tibial nerves and MEP measures, on the other hand, did not reveal any significant differences following training, nor did the extent of changes in these excitability measures relate to the time since injury, functional walking parameters, or muscle strength (LEMS).

### BWSTT and plasticity in SCI

BWSTT is a locomotor training technique that can be varied with manual assistance, functional electrical stimulation or even robot assistance, as used in this study with the HAL® exoskeleton. Functional improvements could be demonstrated in our pilot study with eight SCI patients, where functional examinations of seven of the 11 patients (cases 1–7) were reported, and in an additional single case report [28, 29]. Functional data from the remaining four patients (cases 8–11), as well as electrophysiological data of all 11 patients were added here. Comparing different therapeutic interventions, a meta-analysis including eight studies with SCI patients demonstrated no superiority of one type of assistance training [43], however plastic effects in S1 or M1 of different locomotor-based therapies have not yet been systematically investigated. One case study with four SCI patients demonstrated brain plasticity after 12 weeks of robotic BWSTT with Lokomat® (Hocoma, Switzerland) [44]. The Lokomat® is a driven walking orthesis that is synchronized to the treadmill speed and allows the robotic device to guide the legs through a programmed walking pattern. All patients had greater BOLD response in the foot and leg areas of M1 and S1 and in cerebellum when performing ankle plantar flexion and toe flexion using task-related functional magnetic resonance tomography. Only those patients with substantially increased cerebellar activation, however, demonstrated an improvement in their ability to walk over ground. The authors postulated that cerebellar activation was necessary for feedback loop monitoring of peripheral afferent impulses generating appropriate locomotor pattern. This study showed that one mechanism of brain plasticity due to BWSTT is the renewal of lower extremity representation in S1 and M1. Our electrophysiological data is consistent with this fMRI data, both supporting an effect of BWSTT on cortical responsiveness. Another interesting result of this case study is that an enlargement of S1 representation of lower extremities accompanied the enlargement of M1 activation. This could be related to the walking training itself, which requires intensive afferent feedback to S1, or it could be the result of new corticocortical connections between M1 and S1 in corresponding representation areas [45]. *Schabrun et al.* demonstrated a positive correlation between S1 and M1 excitability after sensory, peripheral electrical stimulation, suggesting that the changes measured here in our study in S1 may also apply to M1. Interestingly, Knikou et al. investigated motor excitability changes in SCI patients at different levels (spinal, pre- and postsynaptic) following locomotor training. They demonstrated that locomotor training is an effective training method even in chronic SCI that promotes presynaptic inhibition in a phase-dependent manner and restores spinal reflex circuits. That led to normalized contraction and coordination of agonistic and antagonistic muscles of the legs especially in the more impaired leg [46, 47]. Looking at our pilot study, we demonstrated significant functional motor improvements in over-ground walking without exoskeleton assistance after 3 months of BWSTT with HAL® exoskeleton [28]. Combined with the present results, we suggest that walking improvements may be related to a renewed S1 and M1 representation of impaired/lower extremities likely related to the recruitment and more effective use of remaining somatosensory afferent pathways and corticospinal tracts.

As in Winchester’s study, our training effects were independent of the time since injury. Although patients whose injury is more recent are thought to have a greater “plastic capacity”, our data do not support such a dependency on walking improvement following training. This indicates that other neuronal instances than the spinal cord itself may be integral in functional improvement. Supraspinal control, for example, has been shown to be necessary for bipedal walking in humans, unlike that found in animals that rely on quadrupedal locomotion [31].

Recent evidence supports a role of spinal circuitry in motor-complete SCI. [48, 49]. The authors report that spinal circuitries distal to the lesion can be used to generate voluntary-controlled motor tasks when they are adequately activated, e. g., by sensory input [50], thereby arguing for a role of intra-spinal sensory-motor feedback [51, 52]. The spinal cord receives sensory information and can make a decision as to what the appropriate response is at that time during the locomotion cycle [51, 52]. This automaticity can be involved in walking rehabilitation techniques like treadmill training with a highly repetition. Combined with supraspinal recruiting of corticospinal and extrapyramidal tracts, brain and spinal instances can activate motoneurons to generate force and speed [53, 54]. For these fine adjustments, it seems to be obvious that not only supraspinal areas but also spinal circuitry may also play a key role in the rehabilitation of locomotion, and with exoskeleton training.

In our study, however, we investigate only patients with motor-incomplete paraplegia. Therefore, a portion of the cortico-spinal connections were preserved in these patients, making is likely that these supraspinal connections indeed play a role in the rehabilitation. This is supported by the fact that motor-incomplete patients show better recovery than motor-complete patients [50]. Nonetheless, we cannot discount the importance of internal spinal circuits, as this is not specifically tested by the measures used here (e.g., ENG, SEP, and MEP). Changes on the spinal level that could have been activated during BWSTT with HAL include neuronal sprouting, reduced spinal excitability, altered ion channel expression [55, 56], and the up-regulation of growth factors related to cell survival, synaptic plasticity, and excitability (e. g., BDNF) [23, 57]. Therefore, we postulate that both supraspinal as well as spinal connections play a key role in the rehabilitation of locomotion, and in particular in exoskeleton training.

Nevertheless, in our study, we have to take into account critically that all our patients had motor-incomplete paraplegia. That means that all had a better functional recovery than motor-complete SCI patients and that the important and crucial supraspinal control effect on spinal circuitries is partly preserved [58]. This may have led to the observation that supraspinal instances have mainly driven therapeutic effect. Thus, we cannot exclude completely a spinal origin of adaptions of the nervous system. The unchanged results of ENG, SEP of tibial nerves and MEP prior to and after training do not indicate sprouting of neurons on spinal level, which might be one mechanism. Moreover, the time interval of 3 months would be too short for a measurable and visible effect on standard electrophysiological parameter. All these changes and mechanisms might have been activated during BWSTT with HAL and have to be considered as possible effects to have influence the functional improvements additionally to supraspinal mechanisms.

### S1-plasticity in human SCI

One novel finding from our study is that we directly show changes in excitability in S1 representations in human SCI following to BWSTT with HAL®. In animals, *Kao et al.* analyzed effects of treadmill locomotion on somatotopic S1organization of forelimb and hindlimb after thoracic transection in adult rats [59, 60]. They demonstrated an increased excitability in both representation areas, both in paralytic hindlimb and preserved forelimb, after the training period. They proposed that various preconditions in rats and humans lead to these results. Considering non-impaired forelimbs, rats showed decreased response to peripheral stimuli at baseline due to immobilization. Therefore, coming from this lowered activity level, authors proposed that BWSTT lead to an increased excitability. In humans, several studies in chronic SCI patients demonstrated an increased excitability in motor and somatosensory pathways proximal to the spinal lesion, which is likely dedicated to optimizing functional adaptions to a new state of external and internal conditions after SCI.

Excitability measurements in M1 or S1 in patients with SCI are of interest to evaluate cortical plasticity and are in general feasible using standardized and established methods like paired-pulse MEP- and SEP-protocols [13, 17, 61]. Due to high-grade spinal cord lesions, however, we were unable to asses many of these parameters in regions caudal to the lesion (i. e. legs), and thus in those areas showing paralysis and sensory loss, in most of the patients. We could, however, derive motor and sensory pathways rostral to the spinal cord lesion, therefore [4], in our study, we decided to assess cortical excitability in S1 by means of ppSEP of median nerves, knowing that only an indirect conclusion to plastic changes in the cortical representation of impaired extremities would be possible. Another critical point is that our control group was younger than the patients group. Instead, we do not expect age-related effects on amplitude ratio in this case, while Lenz et al. demonstrated stable amplitude ratios until the age of 55 years [62]. However, in older adults (60–80 years), paired-pulse suppression is significantly reduced in S1 with amplitude ratios > 1. In their study, Lenz and coworkers linked changes of excitability to tactile discrimination behavior. They found that lack of paired-pulse suppression was associated with impaired tactile perceptual abilities. In our recent study, we used paired-pulse suppression only as a marker of altered cortical excitability. In further studies, linking changes of excitability to behavior might be an interesting issue. Finally, the last limitation is that we did not register the thresholds and current values systematically in this study. However, all stimulations independently from SCI or HC were supramaximal. Our previous study showed that a stimulation intensity of 250 % of sensory threshold or slightly above the motor threshold should be used [18].

### Other supraspinal foci for plasticity

Even though all patients showed functional improvements and excitability changes, there was no significant correlation between functional and electrophysiological parameters. As indicated by normalized S1 excitability following training, cortical reorganization in S1 seems to play an important role in BWSTT with HAL® exoskeleton. It does not appear, however, to be the crucial mechanism driving functional walking improvement. It is known that the cerebellum serves as an important focus of neural plasticity in response to locomotor training [44]. It coordinates normal movements and contributes to motor adaption and motor learning via input from different neuronal systems, adjusting its output accordingly [63, 64]. Cerebellar outputs can influence spinal cord walking control centers, M1, brainstem nuclei, and basal ganglia directly, and has been shown to play an essential role in so called predictive (i.e., feedforward) locomotor adjustments [65]. This concept maintains that the cerebellum enables the body to adapt to predictable but not to sudden, unpredictable motor changes. Predictive adjustments require practice and result in the storage of new movement patterns. To demonstrate the cerebellar function on locomotion, *Morton and Bastian* used a split-belt treadmill on healthy controls and patients with cerebellar disorders [65]. They could show that cerebellar damage lead to impairment of predictive feedforward, but not of reactive motor adaptation. HAL® exoskeleton-assisted BWSTT represents a typical feedforward, predictive locomotor adaption training method. It is not based on quick responses to ongoing afferent feedback [65, 66]. Taking this into consideration, it seems to be conclusive that neuronal plasticity in certain regions of the cerebellum is crucial for the functional improvement we observed. This assumption goes along with Winchester’s observations that only those motor-incomplete patients who demonstrated a substantial increase in cerebellar activation regained the ability to walk over-ground [44].

## Conclusion

In conclusion, these findings suggest that HAL®-assisted BWSTT can induce neuronal plasticity in the primary somatosensory cortex. We did not find a correlation between functional abilities for over-ground walking and changes of excitability in S1, suggesting that either other cortical areas or even a complex supraspinal network is required for walking rehabilitation. Further neuroimaging studies on SCI patients will shed light on the complex field of neuronal plasticity in walking rehabilitation.

## Abbreviations

ASIA: American Spinal Injury Association
BDNF: Brain derived neurotrophic factor
BOLD: Blood-oxygen-level dependent
BWSTT: Body weight supported treadmill training
CMAP: Compound muscle action potential
CMCT: Central motor conduction time
EMG: Electromyography
ENG: Electroneurography
HAL®: Hybrid assistive limb®
HC: Healthy controls
Hz: Hertz
mCV: Motor conduction velocity
MEP: Motor evoked potential
ms: Milliseconds
M1: Primary motor cortex
NA: Not applicable
NR: No response
PMCT: Peripheral motor conduction time
ppSEP: Paired-pulse somatosensory evoked potentials
SCI: Spinal cord injury
sCV: Sensory conduction velocity
SEM: Standard error of the mean
SEP: Somatosensory evoked potentials
SNAP: Sensory nerve action potential
S1: Primary somatosensory cortex
TMS: Transcranial magnetic stimulation
ZPP: Zone of partial preservation

## References

[1] Cohen LG, Roth BJ, Wassermann EM, Topka H, Fuhr P, Schultz J (1991). Magnetic stimulation of the human cerebral cortex, an indicator of reorganization in motor pathways in certain pathological conditions. J Clin Neurophysiol.

[2] Levy WJ, Amassian VE, Traad M, Cadwell J (1990). Focal magnetic coil stimulation reveals motor cortical system reorganized in humans after traumatic quadriplegia. Brain Res.

[3] Laubis-Herrmann U, Dichgans J, Bilow H, Topka H (2000). Motor reorganization after spinal cord injury: evidence of adaptive changes in remote muscles. Restor Neurol Neurosci.

[4] Topka H, Cohen LG, Cole RA, Hallett M (1991). Reorganization of corticospinal pathways following spinal cord injury. Neurology.

[5] Lotze M, Laubis-Herrmann U, Topka H, Erb M, Grodd W (1999). Reorganization in the primary motor cortex after spinal cord injury—A functional Magnetic Resonance (fMRI) study. Restor Neurol Neurosci.

[6] Henderson LA, Gustin SM, Macey PM, Wrigley PJ, Siddall PJ (2011). Functional reorganization of the brain in humans following spinal cord injury: evidence for underlying changes in cortical anatomy. J Neurosci.

[7] Humanes-Valera D, Aguilar J, Foffani G (2013). Reorganization of the intact somatosensory cortex immediately after spinal cord injury. PLoS One.

[8] Jurkiewicz MT, Mikulis DJ, McIlroy WE, Fehlings MG, Verrier MC (2007). Sensorimotor Cortical Plasticity During Recovery Following Spinal Cord Injury: A Longitudinal fMRI Study. Neurorehabil Neural Repair.

[9] Streletz LJ, Belevich JK, Jones SM, Bhushan A, Shah SH, Herbison GJ (1995). Transcranial magnetic stimulation: cortical motor maps in acute spinal cord injury. Brain Topogr.

[10] Curt A, Alkadhi H, Crelier GR, Boendermaker SH, Hepp-Reymond M-C, Kollias SS (2002). Changes of non-affected upper limb cortical representation in paraplegic patients as assessed by fMRI. Brain.

[11] Rijntjes M, Tegenthoff M, Liepert J, Leonhardt G, Kotterba S, Müller S (1997). Cortical reorganization in patients with facial palsy. Ann Neurol.

[12] Pons TP, Garraghty PE, Ommaya AK, Kaas JH, Taub E, Mishkin M (1991). Massive cortical reorganization after sensory deafferentation in adult macaques. Science.

[13] Kujirai T, Caramia MD, Rothwell JC, Day BL, Thompson PD, Ferbert A (1993). Corticocortical inhibition in human motor cortex. J Physiol.

[14] Rosenkranz K, Williamon A, Rothwell JC (2007). Motorcortical Excitability and Synaptic Plasticity Is Enhanced in Professional Musicians. J Neurosci.

[15] Shagass C, Schwartz M (1964). Recovery functions of somatosensory peripheral nerve and cerebral evoked responses in man. Electroencephalogr Clin Neurophysiol.

[16] Ragert P, Dinse HR, Pleger B, Wilimzig C, Frombach E, Schwenkreis P (2003). Combination of 5 Hz repetitive transcranial magnetic stimulation (rTMS) and tactile coactivation boosts tactile discrimination in humans. Neurosci Lett.

[17] Höffken O, Veit M, Knossalla F, Lissek S, Bliem B, Ragert P (2007). Sustained increase of somatosensory cortex excitability by tactile coactivation studied by paired median nerve stimulation in humans correlates with perceptual gain. J Physiol.

[18] Höffken O, Tannwitz J, Lenz M, Sczesny-Kaiser M, Tegenthoff M, Schwenkreis P (2013). Influence of parameter settings on paired-pulse-suppression in somatosensory evoked potentials: A systematic analysis. Clin Neurophysiol.

[19] Hoshiyama M, Kakigi R (2002). New concept for the recovery function of short-latency somatosensory evoked cortical potentials following median nerve stimulation. Clin Neurophysiol.

[20] Lenz M, Höffken O, Stude P, Lissek S, Schwenkreis P, Reinersmann A (2011). Bilateral somatosensory cortex disinhibition in complex regional pain syndrome type I. Neurology.

[21] Ragert P, Franzkowiak S, Schwenkreis P, Tegenthoff M, Dinse HR (2008). Improvement of tactile perception and enhancement of cortical excitability through intermittent theta burst rTMS over human primary somatosensory cortex. Exp Brain Res.

[22] Protas EJ, Holmes SA, Qureshy H, Johnson A, Lee D, Sherwood AM (2001). Supported treadmill ambulation training after spinal cord injury: a pilot study. Arch Phys Med Rehabil.

[23] Macias M, Nowicka D, Czupryn A, Sulejczak D, Skup M, Skangiel-Kramska J (2009). Exercise-induced motor improvement after complete spinal cord transection and its relation to expression of brain-derived neurotrophic factor and presynaptic markers. BMC Neurosci.

[24] de Leon RD, Tamaki H, Hodgson JA, Roy RR, Edgerton VR (1999). Hindlimb locomotor and postural training modulates glycinergic inhibition in the spinal cord of the adult spinal cat. J Neurophysiol.

[25] Girgis J, Merrett D, Kirkland S, Metz GAS, Verge V, Fouad K (2007). Reaching training in rats with spinal cord injury promotes plasticity and task specific recovery. Brain.

[26] Krajacic A, Weishaupt N, Girgis J, Tetzlaff W, Fouad K (2010). Training-induced plasticity in rats with cervical spinal cord injury: effects and side effects. Behav Brain Res.

[27] Kubota S, Nakata Y, Eguchi K, Kawamoto H, Kamibayashi K, Sakane M (2013). Feasibility of rehabilitation training with a newly developed wearable robot for patients with limited mobility. Arch Phys Med Rehabil.

[28] Aach M, Cruciger O, Sczesny-Kaiser M, Höffken O, Meindl RC, Tegenthoff M (2014). Voluntary driven exoskeleton as a new tool for rehabilitation in chronic spinal cord injury: a pilot study. Spine J.

[29] Cruciger O, Tegenthoff M, Schwenkreis P, Schildhauer TA, Aach M (2014). Locomotion training using voluntary driven exoskeleton (HAL) in acute incomplete SCI. Neurology.

[30] Krakauer JW (2006). Motor learning: its relevance to stroke recovery and neurorehabilitation. Curr Opin Neurol.

[31] Nielsen JB (2003). How we walk: central control of muscle activity during human walking. Neuroscientist.

[32] Kawamoto H, Kamibayashi K, Nakata Y, Yamawaki K, Ariyasu R, Sankai Y (2013). Pilot study of locomotion improvement using hybrid assistive limb in chronic stroke patients. BMC Neurol.

[33] Dinse HR (2012). Long-term sensory stimulation therapy improves hand function and restores cortical responsiveness in patients with chronic cerebral lesions. Three single case studies. Front Hum Neurosci.

[34] Waters RL, Adkins R, Yakura J, Vigil D (1994). Prediction of ambulatory performance based on motor scores derived from standards of the American Spinal Injury Association. Arch Phys Med Rehabil.

[35] Guideline thirteen: guidelines for standard electrode position nomenclature (1994). American Electroencephalographic Society. J Clin Neurophysiol.

[36] Guideline nine: guidelines on evoked potentials (1994). American Electroencephalographic Society. J Clin Neurophysiol.

[37] Schwenkreis P, Tegenthoff M. Evozierte Potenziale in der Diagnostik spinaler Erkrankungen. Klin Neurophysiol. 2005.

[38] van Hedel HJ, Wirz M, Dietz V (2005). Assessing walking ability in subjects with spinal cord injury: validity and reliability of 3 walking tests. Arch Phys Med Rehabil.

[39] van Hedel HJA, Wirz M, Dietz V (2008). Standardized assessment of walking capacity after spinal cord injury: the European network approach. Neurol Res.

[40] Podsiadlo D, Richardson S (1991). The timed “Up & Go”: a test of basic functional mobility for frail elderly persons. J Am Geriatr Soc.

[41] Harada ND, Chiu V, Stewart AL (1999). Mobility-related function in older adults: assessment with a 6-min walk test. Arch Phys Med Rehabil.

[42] Piepmeier JM, Jenkins NR (1988). Late neurological changes following traumatic spinal cord injury. J Neurosurg.

[43] Morawietz C, Moffat F (2013). Effects of locomotor training after incomplete spinal cord injury: a systematic review. Arch Phys Med Rehabil.

[44] Winchester P, McColl R, Querry R, Foreman N, Mosby J, Tansey K (2005). Changes in supraspinal activation patterns following robotic locomotor therapy in motor-incomplete spinal cord injury. Neurorehabil Neural Repair.

[45] Schabrun SM, Ridding MC, Galea MP, Hodges PW, Chipchase LS (2012). Primary sensory and motor cortex excitability are co-modulated in response to peripheral electrical nerve stimulation. PLoS One.

[46] Knikou M, Mummidisetty CK (2014). Locomotor training improves premotoneuronal control after chronic spinal cord injury. J Neurophysiol.

[47] Knikou M (2010). Neural control of locomotion and training-induced plasticity after spinal and cerebral lesions. Clin Neurophysiol.

[48] Dietz V, Grillner S, Trepp A, Hubli M, Bolliger M (2009). Changes in spinal reflex and locomotor activity after a complete spinal cord injury: a common mechanism?. Brain.

[49] Thompson AK, Wolpaw JR (2014). Operant conditioning of spinal reflexes: from basic science to clinical therapy. Front Integr Neurosci.

[50] Edgerton VR, Roy RR. The nervous system and movement. In: ACSM’s Advanced Exercise Physiology, edited by Tipton CM. Philadelphia: Lippincott Williams & Wilkins, 2006, p. 41–94.

[51] Edgerton VR, Leon RD, Harkema SJ, Hodgson JA, London N, Reinkensmeyer DJ (2001). Retraining the injured spinal cord. J Physiol.

[52] Hodgson JA, Roy RR, de Leon R, Dobkin B, Edgerton VR (1994). Can the mammalian lumbar spinal cord learn a motor task?. Med Sci Sports Exerc.

[53] Edgerton VR, Roy RR (2012). A new age for rehabilitation. Eur J Phys Rehabil Med.

[54] Solopova IA, Selionov VA, Sylos-Labini F, Gurfinkel VS, Lacquaniti F, Ivanenko YP (2015). Tapping into rhythm generation circuitry in humans during simulated weightlessness conditions. Front Syst Neurosci.

[55] Fouad K, Rank MM, Vavrek R, Murray KC, Sanelli L, Bennett DJ (2010). Locomotion after spinal cord injury depends on constitutive activity in serotonin receptors. J Neurophysiol.

[56] Boulenguez P, Liabeuf S, Bos R, Bras H, Jean-Xavier C, Brocard C (2010). Down-regulation of the potassium-chloride cotransporter KCC2 contributes to spasticity after spinal cord injury. Nat Med.

[57] Boyce VS, Tumolo M, Fischer I, Murray M, Lemay MA (2007). Neurotrophic factors promote and enhance locomotor recovery in untrained spinalized cats. J Neurophysiol.

[58] Wirz M, Colombo G, Dietz V (2001). Long term effects of locomotor training in spinal humans. J Neurol Neurosurg Psychiatry.

[59] Kao T, Shumsky JS, Murray M, Moxon KA (2009). Exercise induces cortical plasticity after neonatal spinal cord injury in the rat. J Neurosci.

[60] Kao T, Shumsky JS, Knudsen EB, Murray M, Moxon KA (2011). Functional role of exercise-induced cortical organization of sensorimotor cortex after spinal transection. J Neurophysiol.

[61] Klostermann F, Funk T, Vesper J, Siedenberg R, Curio G (2000). Double-pulse stimulation dissociates intrathalamic and cortical high-frequency (>400Hz) SEP components in man. Neuroreport.

[62] Lenz M, Tegenthoff M, Kohlhaas K, Stude P, Höffken O, Gatica Tossi MA (2012). Increased excitability of somatosensory cortex in aged humans is associated with impaired tactile acuity. J Neurosci.

[63] Pisotta I, Molinari M (2014). Cerebellar contribution to feedforward control of locomotion. Front Hum Neurosci.

[64] Christensen LO, Johannsen P, Sinkjaer T, Petersen N, Pyndt HS, Nielsen JB (2000). Cerebral activation during bicycle movements in man. Exp Brain Res.

[65] Morton SM, Bastian AJ (2006). Cerebellar contributions to locomotor adaptations during splitbelt treadmill walking. J Neurosci.

[66] Shadmehr R, Smith MA, Krakauer JW (2010). Error correction, sensory prediction, and adaptation in motor control. Annu Rev Neurosci.

